# Tracking, naming, specifying, and comparing implementation strategies for person-centred care in a real-world setting: a case study with seven embedded units

**DOI:** 10.1186/s12913-022-08846-x

**Published:** 2022-11-24

**Authors:** Helena Fridberg, Lars Wallin, Malin Tistad

**Affiliations:** 1 School of Health and Welfare, Dalarna University, Falun, Sweden; 2 Institute of Health and Care Sciences and University of Gothenburg Centre for Person-Centred Care, Sahlgrenska Academy at the University of Gothenburg, Gothenburg, Sweden; 3 Department of Neurobiology, Care Sciences and Society, Karolinska Institute, Stockholm, Sweden

**Keywords:** Implementation strategies, Reporting, Person-centred care, Case study

## Abstract

**Background:**

The implementation of person-centred care (PCC) is advocated worldwide. Stakeholders in charge of implementing PCC as a broad-scale change across the health care sector face two intertwined and complex challenges. First, making sense of PCC as an intervention with complex innovation characteristics and second, staging implementation of PCC by choosing appropriate implementation strategies. We aimed to explore one of these challenges by tracking, naming, specifying, and comparing which strategies and how strategies were enacted to support the implementation of more PCC in a real-world setting represented by one health care region in Sweden.

**Methods:**

A case study with seven embedded units at two organisational levels within a health care region was conducted from 2016 to 2019. Data were collected from three sources: activity logs, interviews, and written documents. Strategies were identified from all sources and triangulated deductively by name, definition, and cluster in line with the taxonomy Expert Recommendations for Implementing Change (ERIC) and specified according to recommendations by Proctor and colleagues as actor, action, action target, temporality, dose, outcome, and justification.

**Results:**

Four hundred thirteen activities were reported in logs, representing 43 discrete strategies identified in ERIC (*n* = 38), elsewhere (*n* = 1), or as emerging strategies (*n* = 4). The highest reported frequencies of discrete strategies were identified as belonging to two clusters: Train and educate stakeholders (40%) and Develop stakeholder interrelationships (38%). We identified a limited number of strategies belonging to the cluster Use evaluative and iterative strategies (4.6%) and an even smaller number of strategies targeting information to patients about the change initiative (0.8%). Most of the total dose of 11,076 person-hours in the 7 units was spent on strategies targeting health care professionals who provide PCC (81.5%) while the dose of strategies targeting support functions was 18.5%.

**Conclusions:**

Our findings show both challenges and merits when strategies for implementation of PCC are conducted in a real-world setting. The results can be used to support and guide both scientists and practitioners in future implementation initiatives.

**Supplementary Information:**

The online version contains supplementary material available at 10.1186/s12913-022-08846-x.

## Background

### Implementation

Implementation efforts performed in real-world settings across the health care sector rely on various stakeholders to use diverse activities to drive the change [1]. The selection of activities to support the change is often based on these stakeholders’ intuition and experience and seldom based on, enacted or reported in line with theory, empirical evidence, or recommended guidelines [2]. The activities contain the active “how to” in implementation research and practice and are denoted strategies [3]. Implementation strategies can be defined as “methods or techniques used to improve adoption, implementation, sustainment, and scale-up of interventions” [3]. Implementation strategies are considered integral in implementation efforts and in improving the understanding of successful implementation initiatives [3–5].

Tracking and reporting strategies rigorously and in sufficient detail and assessing their practicality has been listed as one of the top five priorities in research for enhancing the interpretation of the impact of implementation strategies [6]. Recent research initiatives have sought to fill several identified gaps regarding tracking and reporting implementation strategies [2, 7–9]. Recommendations include naming strategies by adhering to taxonomies with consistent terminology [5] and defining what each strategy entails [3]. Researchers are also recommended to adhere to methodologies that enhance reporting of the dose and tracking of implementation strategies across time [7]. Moreover, previous research has recommended triangulating data from multiple sources to identify and describe discrete strategies in implementation efforts [10]. While these recommendations focus on the description and reporting of strategies, another challenge that researchers and clinicians face is which strategies to select for a particular innovation in relation to its specific context [11]. Knowledge on choosing the most appropriate strategies is still lacking and even implementation scientists struggle to match strategies to identified barriers [12]. Moreover, while selecting appropriate strategies is a complex task, the challenge becomes even greater if the innovation to be implemented is perceived as complex [13].

### Person-centred care - a complex innovation

Politicians, policymakers, researchers, patients (representative organisations), and health care professionals (HCPs) globally are calling for a change whereby patients should no longer be seen as passive reciprocates of health care but instead as active participants [14]. Recognising patients’ aspirations, wishes, and needs for health care should be a natural aspect in care settings and a prerequisite for delivering high-quality care [14]. Person-centred care (PCC) has been advanced as a concept of achieving this paradigm shift [1, 15, 16]. PCC embraces the ethical position that each patient is unique and needs to be approached and treated accordingly [16, 17]. Each person’s uniqueness is based on their lived experiences, formed by the contexts they have lived and live in and the relationships they have formed and encountered throughout their lives [17–19]. PCC has been perceived as a highly complex innovation by HCPs and stakeholders [1, 20]. Thus, to achieve the paradigm shift that involves a broad scale change permeating the whole care sector is a challenging enterprise [1, 21, 22].

### Implementation of PCC in a real-world setting

Implementation of PCC as a broad-scale change across health care settings has not been studied at length. Furthermore, the literature lacks information about the inherent challenges addressed in real-world settings without the support of researchers. Research on implementation in real-world settings can increase our understanding of strategies chosen from a bottom-up perspective. Observing and evaluating strategies chosen and enacted in a real-world setting can point to weaknesses and strengths of the effort and thus improve future research and implementation initiatives of similar innovations in comparable contexts [23]. Therefore, the study aims to increase the knowledge on which strategies are applied and how they are used to support implementation of PCC in a real-world setting.

## Methods

An embedded case study design [24] was used to explore the strategies enacted to support the implementation of more PCC across different health care specialities within a health care organisation.

### The case

The case is defined as *strategies to support the implementation of PCC*. The implementation is conducted “as usual,” i.e., as part of current implementation efforts without involvement from researchers. In September 2015, political leaders in the region took a policy decision to initiate a transition towards more PCC. The Department for Development (DD) staff, with a primary task of supporting development and improvement work in the region, was assigned to lead the implementation initiative. This assignment created an opportunity to study a case in which a health care region was observed longitudinally to explore and compare which and how strategies were enacted to support the implementation of more PCC.

### Setting

This study took place in Sweden, where most health care regions have resolved to implement more PCC to improve health care [25, 26].

In Sweden, health care is publicly funded, and the country is divided into 21 regions responsible for the provision of health care to all inhabitants. While regions need to adhere to Swedish health care laws (e.g., provide care in line with evidence and within a set time), they have considerable freedom to decide how to organise health care to fit their local conditions.

**Fig. 1:**
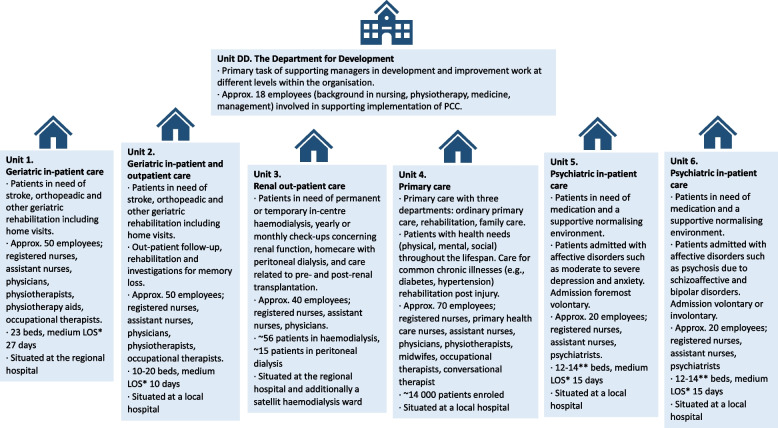
The Department for Development and six healthcare units each represent one of the case’s embedded units. * LOS: Length of stay, including potential temporary leave. ** Units 5 and 6 were merged between June 2016 and August 2018. Eighteen beds were available during that period

In this study we collected data in one region in central Sweden. At the time of the study, the region was provided one large regional hospital, five smaller local hospitals, and approximately 30 primary health care units supporting 280,000 inhabitants spanning an area of 28,000 km^2^. We recruited 7 units at two organisational levels within the health care region. One overarching unit, the region’s DD, and six health care units participated in the study. The DD supported the transition to more PCC across the region. The health care units were a convenience sample based on units (*n* = 11) that participated in a first PCC teaching initiative arranged by the DD staff, diversity of care provided at the region, and senior and frontline managers’ approval to be part of the study. The units are depicted in Fig. 1. Units 5 and 6 were merged due to a staff shortage shortly after the first learning initiative. However, they wished to be included as separate units as they aimed to keep their HCPs working within their original teams.

**Fig. 2:**
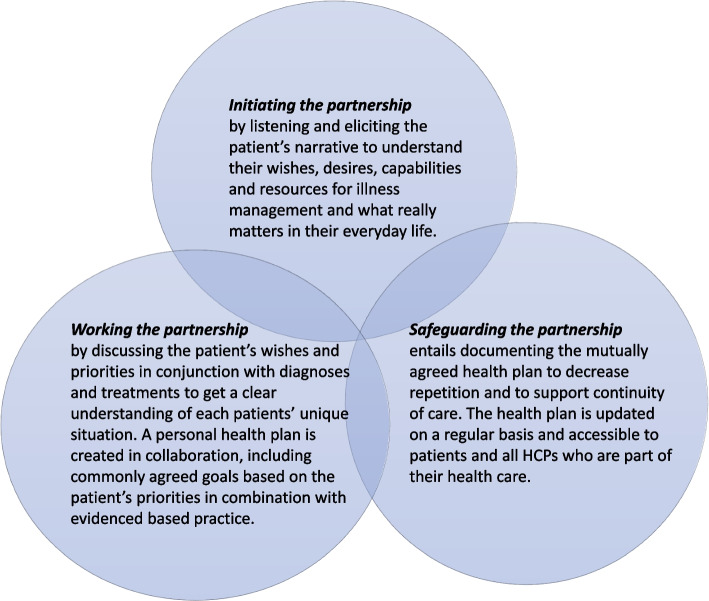
The three clinical routines proposed by the GPCC to support clinicians achieve more person-centred care in their daily work

Staff at the DD chose to introduce PCC (the innovation) in line with the model put forward by The Gothenburg University Centre for Person-centred Care (GPCC) [15, 27]. Researchers at GPCC underscore the ethical underpinnings of PCC at the same time as they have introduced three routines to aid clinicians to achieve more PCC in their practice [15, 28]. These routines are based on initiating, working, and safeguarding the partnership between patients and HCPs (see Fig. 2).

### Participants

Staff in charge of supporting implementation of PCC at the DD and the six health care units (here denoted as change agents) participated in interviews. Separate interviews were held at each unit and the number of participants in each interview was based on who was considered a change agent at that specific unit. In general, these change agents who had volunteered to be part of the change initiative were asked to participate as they represented different care specialties at their unit or were seen to possess valuable knowledge from quality improvement work in line with their existing roles (e.g., quality developers).

Change agents at the DD and units 1 and 2 participated in dyadic interviews [29] and change agents from units 3 to 6 participated in focus groups [30] (see Table 1 for participant characteristics).

**Table 1:** Number of change agents participating in interviews at each unit and their vocational roles

Unit	n	Vocational role
DD	2	Quality developers
1	2	Frontline manager Assistant frontline manager
2	2	Frontline manager Quality developer
3	6	Frontline manager Assistant frontline manager HCPs represented by different care specialities at the unit: nephrology investigations and check-ups, haemo, peritoneal, and home dialysis.
4	6	Senior manager Quality developer Frontline managers representing three departments: Ordinary primary care, family centre, and rehabilitation
5	4	Frontline manager Coordination nurse Registered nurse Assistant nurse
6	4	Frontline manager Coordination nurse Registered nurse Assistant nurse

### Data sources

We collected data during several stages from three sources: activity logs, interviews, and written documents, i.e., reports, plans, and timetables for activities. First, we collected data on activities conducted at the DD representing a pre-implementation phase lasting from October 2015 until May 2016. These data were collected retrospectively based on recall from the change agents at the DD along with written documents from this phase. Data were not provided for dose and exact temporality as it was impossible for change agents at the DD to estimate these parameters accurately. Second, activity logs were collected retrospectively from all units between May 2016 and May 2017. Data were based on recall from change agents using calendars, documents, and interviews to report enacted activities retrospectively. Third, data were collected prospectively between May 2017 and May 2019 for units 1 to 4 and between May 2017 and November 2019 for units 5 and 6 and the DD. Interviews were conducted on two occasions a year apart at each embedded unit, starting in June 2017 for the DD and at health care units 1 to 4. Interviews for units 5 and 6 started about half a year later owing to organisational changes. Data reported in this study were validated for its content by representatives at each unit who were given access to the summarised activity logs and asked at numerous occasions during the triangulation process to clarify or correct data that were difficult to interpret, unclear, or contradictory.

#### Procedure interviews

The interviews were conducted in a secluded room designated by the manager at each workplace. The last author (MT) functioned as moderator during the first round of interviews while the first author (HF) took on this role during the second round. All authors served as notetakers. A semi-structured interview protocol was used (see Additional file 1), focusing on questions covering activities and strategies to support the change towards more PCC. The questions were sent in advance so that participants could prepare for the interviews. The moderator/interviewer used member summaries throughout the interviews to validate her interpretation [31]. When interviews were conducted a second time, an overview was made of what had been said on the first occasion and participants could comment and critique what was said. The interview was then followed by what had occurred during the past year. Interviews ranged from 41 to 99 minutes (mean 71), were recorded, and transcribed verbatim.

#### Procedure activity logs

We developed an electronic activity log in line with previous work and recommendations by Bunger et al. [7]. Change agents were assigned to use this log to report all implementation activities enacted to achieve more PCC [2, 7]. Reports were focused on naming and describing the type of activity being enacted, its purpose, who was running the activity and how many participants were involved, date and time consumption, and clarifying comments from the change agents who kept the activity logs [3]. Activities of short duration that were repeated frequently, such as “small chats”, were encouraged to be estimated weekly. Because this study targeted implementation in a real-world setting, we did not give any guidance regarding taxonomies that could have been used to choose or describe implementation strategies in the activity logs [32]. This choice was taken to decrease the risk of influencing change agents to use strategies they had not previously thought of using.

The change agents were encouraged to report on a weekly to monthly basis to minimise recollection bias. However, some change agents had difficulties adhering to this recommendation due to a high workload and were instead encouraged to use their calendars on a half-year basis to track their implementation strategies. In some instances, representatives from the research group met with change agents and helped to fill out the logs. Changes were made in the summarised activity logs if change agents identified any corrections that had to be made to give an accurate representation of the activities performed.

#### Data collection documents

We collected documents related to activities passed to implement PCC at the units. Documents ranged from formal reports from the DD representatives to policymakers in the region, to plans and timetables for learning seminars, meetings, and workshops (e.g., types of activity performed and targets for the activity).

### Development of a coding manual

A coding manual was developed in which identified strategies were deductively named and defined according to the Expert Recommendations for Implementing Change (ERIC) taxonomy [5]. ERIC contains 73 named and defined discrete strategies. We considered other taxonomies for our coding of strategies [33–37], and ERIC was chosen because it has been used extensively in health care settings and it was seen to fit with the level of detail in our collected data (e.g., scarce information on conceptual action targets precluded use of taxonomies requiring granular information on behaviour change) [36]. Moreover, using a well-known taxonomy such as ERIC enhances a consistent vocabulary which enables comparisons across implementation research initiatives [5, 38]. The definitions from ERIC were adapted to the innovation and context. The coding manual included inclusion and exclusion criteria and examples and quotes from different data sources to facilitate coding and increase the trustworthiness of the data [39]. In addition, we were prepared to include newly proposed strategies from three recent studies in the coding manual [2, 7, 10]. Facilitation was not included in this manual due to our understanding that it would be difficult to separate from many of the other discrete strategies in ERIC [10]. ERIC’s implementation strategies have been subject to concept mapping with cluster analyses. The coding manual was organised in accordance with these nine clusters: use evaluative and iterative strategies, provide interactive assistance, adapt and tailor to context, develop stakeholder relationships, train and educate stakeholders, support clinicians, engage consumers, utilize financial strategies, and change infrastructure [40]. Strategies that could not be mapped to the ERIC taxonomy or the newly proposed strategies were explored, named, and defined individually and subsequently added to the coding manual (see Additional file 2). HF developed the coding manual and the mapping of strategies. The mapping was reviewed and discussed in weekly consensus meetings by all co-authors.

### Data analysis

Analysis was first conducted and summarised in separate templates for each embedded unit. Once the analysis had been finalised for each unit, data were compared across all units and merged to describe the case. The templates were constructed in line with strategy reporting guidelines from Proctor et al. [3]. They outlined name, definition, and cluster according to the ERIC taxonomy [5, 40] and the developed coding manual, along with actor, action, action target, temporality, dose, outcomes affected, and justification as shown in Table 2.

**Table 2:** Overview of strategy characteristics specified using Proctor et al.’s recommendations [3] and data sources and analysis description

Strategy characteristic	Definition/explanation	Data sources and analysis
Name and definition	Name of strategy, its definition^a^, and cluster based on the ERIC taxonomy [5, 40] and the developed coding manual.	Data from all sources^b^ triangulated to name and cluster strategies according to the ERIC. Coding was aided by a developed coding manual adapted to the innovation and context. Identified strategies that did not fit the ERIC taxonomy were individually named and defined. All coding underwent consensus discussions within the research group. Discrete strategies that could be linked to activity logs were summed to report frequencies.
Actor	Person(s) who delivers the strategy	Data primarily identified in activity logs and, in some cases, triangulated with other sources. Actor was described in the text.
Action	Operationalisation of the strategy	Data triangulated from all sources and described in the text.
Action target	The person(s) or factors targeted by the strategy	Data answering the “who” were primarily found in activity logs. The “what,” i.e., the conceptual target, was sometimes identified in interviews and other documents. “Who” and “what” were described in the text. Moreover, two levels of action targets were identified. Reported activities directly targeting HCPs engaged in adopting and integrating PCC in their practice versus strategies targeting change agents and stakeholders supporting the implementation initiative. These two levels were reported concerning frequencies of strategies and dose.
Temporality	Time point/time span for enacting the strategy	Strategies in activity logs were first arranged in chronological order according to date starting in May 2016 and then clustered on a half-year basis from January to June and July to December. Strategies enacted in a pre-implementation phase from October 2015 until May 2016 by the DD were described in the text.
Dose	The aggregated time for each unit spent on clusters of strategies	Quantitative data were primarily identified in activity logs and sometimes used in conjunction with documents from timetables and specifications from actors involved in the strategy. Time was calculated as minutes/hours spent on an implementation strategy and the number of participants involved.
Outcomes affected	Expected outcome of enacted strategies	Data triangulated from all sources and described in the text.
Justification	Reason for choosing specific strategies	Data triangulated from all sources to identify whether the strategy was justified by theory, research, experience, or intuition by change agents. Data were described in the text without the authors inferring any justification.

^a^Definition of strategies was aided using ancillary file 6 that is enclosed with ERIC [5]

^b^Data sources derived from activity logs, interviews, documents, i.e., reports, implementation plans, and timetables for activities

All activity logs were analysed whereby the logged activities were coded to strategies according to the coding manual and strategy characteristics in accordance with the template (Table 2) were identified. Interview transcripts and documents were analysed in NVivo and activities related to the implementation of PCC were sought for along with corresponding strategies and strategy characteristics [41]. The process involved continuous triangulation of all data sources to achieve credibility in the identification of activities and coding of discrete strategies along with strategy characteristics. In cases in which findings in the triangulation of the data sources showed inconsistencies, consensus discussions were made in the research group to understand and solve the discrepancy [42]. If consensus could not be reached or in situations requiring more information, change agents were asked to clarify the discrepancy. Reported activities containing several identified strategies were coded to two or more discrete strategies according to ERIC and the developed coding manual (ERIC strategy list numbers are henceforth denoted with “Eric” and provided within brackets while emerging strategies are given a list number from the coding manual and denoted with “new”) [5]. For example, one activity enacted and reported in the activity log by the DD was the work conducted to include the search term “narrative” in patients’ health care records. This activity was identified to inherit three discrete strategies, i.e., *Change record systems* (Eric 12)*, Conduct cyclical small tests of change* (Eric 14), and *Build a coalition* (Eric 6).

Because the dose in the activity logs was linked to reported activities and not to strategies, the dose was summed on an aggregated level according to the ERIC clusters [40] on a six-month basis. When one reported activity contained several strategies that originated from different clusters, the dose was allocated to the cluster identified to include the most prominent strategy for each activity [40]. Activities and strategies that did not have a clear link to activity logs were described in the text as these data lacked quantitative specifications (e.g., how much time was spent on the activity/strategy and the number of persons engaged in the activity).

## Results

The results on the work done by the DD leading up to the first learning seminar (i.e., the pre-implementation phase) is presented first. This is because change agents at the DD started their work a year ahead of the other units and had a leading role in implementing PCC across the region. Thereafter, the results from all units, including the DD, are presented.

### Pre-implementation phase

While all staff at the DD engaged in working with implementation of PCC, two change agents were explicitly given the task to lead the work to enable and develop a plan to initiate and support this change. New funding was accessed, allowing approximately 32 working hours per week shared between the two change agents (Eric 1). Chosen strategies in this phase were primarily focused on creating a plan for the change initiative across the whole region to put this in action. Change agents increased their knowledge about PCC by taking part in conferences and meetings throughout the country (new 74), reading literature about the topic, and talking to different “experts” in the PCC field (Eric 52). An advisory board with various stakeholders in the region was assembled to input the change initiative during the pre-implementation phase (Eric 64). A large part of the change agents’ time was spent developing stakeholder interrelationships within and outside the region. This process included initiation of contact with stakeholders at a regional and national level to gain and share knowledge and promote network weaving, create buy-in at the regional level, receive feedback on the implementation plans, and build coalitions for the implementation effort (Eric 6, 38, 40, 64). Stakeholders involved in this process included patients, union representatives at a local and national level, HCPs and other staff with different vocational roles at the region, staff at the Swedish Association of Local Authorities and Regions, and change agents with similar assignments in other areas of the country.

By the end of the pre-implementation phase, the support approach chosen and developed to elicit a change towards more PCC in the region was based on a combined top-down and bottom-up approach tailored to fit the context and innovation (Eric 63). The initial top-down approach with a pull system was put in action by inviting all health care units in the region to participate in three full-day learning seminars spread over 6 months. Participation was voluntary and new seminars were then run yearly. Units that participated had to enrol several HCPs, preferably representing different vocational roles to support team buy-in. No reimbursement or financial incentives were given to those units who participated; instead, they were supposed to support the transition towards more PCC within their financial budget. However, the learning seminars were free of charge and included lunch for all participants. Change agents at the DD regarded themselves as instigators offering a support function in which health care units were not forced but invited to take part in this change. One change agent at the DD expressed such an attitude:

*Yes, I mean, the reasoning behind these learning seminars from the beginning, and I think it’s still the case, is that we’ve created places and platforms for teams that have the chance to gain knowledge in terms of research and see what others have done. I’ve also connected it to e-health. But it is still the teams themselves who need to apply, who need to be interested and actively participate. We can only, like, offer a place and say that this exists. It’s free and you’re welcome to apply. But the drive and the work have always come from the managers and the teams themselves. And we stand for that strategy.*

(Dyadic interview, Change agent at the DD)

Using this support approach, the DD also implicitly embedded the discrete strategy of having leadership at the unit level decide to opt-in, thereby considering that implementation of PCC should be prioritised and mandated change (Eric 44).

During and after the learning seminars, a bottom-up approach was initiated whereby the change agents at the health care unit level performed activities to support implementation of PCC according to their perceived needs (Eric 63).

Moreover, change agents at the health care units were encouraged to adapt and operationalise PCC to their context (Eric 51). One of the change agents at the DD described this process in the following way:

*A factor for success is that people within their context can decide how to work with person-centred care. No one has told you exactly how to work at the surgical [ward], medical [ward], or primary care unit. Instead, you should find your own [take]. That is, of course, a challenge as well. But I still believe that it evokes creativity.* (Dyadic interview, Change agents at the DD).

After the learning seminars, change agents at the DD supported those change agents at the unit level who actively sought more help in their implementation effort.

### Activities and discrete strategies enacted at units during the implementation phase

A total of 413 activities were reported in activity logs in all units between May 2016 and November 2019. We identified 39 unique discrete strategies previously described in the ERIC taxonomy [5] or elsewhere [2]. Additionally, four strategies emerged in the data that could not be identified in ERIC. While 41 discrete strategies were identified in the activity logs 782 times, two strategies could not be linked to any activity logs and are thus described without quantitative measures. Examples of identified discrete strategies and their specifications are given in Additional file 3.

### Naming and defining strategies

Of the 413 activity logs, 238 activities contained one discrete implementation strategy, 109 activities included two and 66 activities incorporated three or more (see Table 3 for frequency of discrete strategies and clusters).

**Table 3:** Frequency of discrete implementation strategies and clustered strategies identified in the units’ activity logs (*n* = 782). The number denoted to each strategy in the table are from the ERIC strategy list numbers and list numbers from the developed coding manual. ERIC clusters are written in italics in the table creating headings for the strategies underneath that belong to the cluster

Discrete strategies	f	%
*Use evaluative and iterative strategies*	36	4,6
4. Assess for readiness	1	0,1
5. Audit and provide feedback	1	0,1
14. Conduct cyclical small tests of change	2	0,3
23. Develop a formal implementation blueprint	8	1,0
46. Obtain and use patients’ feedback	1	0,1
56. Purposefully re-examine the implementation	23	2,9
*Provide interactive assistance*	2	0,3
53. Provide clinical supervision	2	0,3
*Adapt and tailor to context*	12	1,5
51. Promote adaptability	11	1,4
63. Tailor strategies	1	0,1
*Develop stakeholder interrelationships*	297	38,0
6. Build a coalition	5	0,6
7. Capture and share local knowledge	27	3,5
17. Conduct local consensus discussions	3	0,4
24. Develop academic partnerships	11	1,4
35. Identify and prepare champions	13	1,7
40. Involve executive boards	2	0,3
48. Organize clinician implementation team meetings	99	12,7
52. Promote network weaving	110	14,1
57. Recruit, designate, and train for leadership	19	2,4
64. Use advisory boards and workgroups	2	0,3
65. Use an implementation advisor	6	0,8
*Train and educate stakeholders*	315	40,3
15. Conduct educational meetings	70	9,0
16. Conduct educational outreach visits	6	0,8
19. Conduct ongoing training	15	1,9
20. Create a learning collaborative	1	0,1
29. Develop educational materials	63	8,1
31. Distribute educational materials	54	6,9
43. Make training dynamic	53	6,8
74. Provide stakeholders the possibility to attend educational meetings^b^	51	6,5
77. Communicate^a^	2	0,3
*Support clinicians*	76	9,7
21. Create new clinical teams	1	0,1
58. Remind clinicians	17	2,2
59. Revise professional roles	8	1,0
75. Provide stakeholders with resources^b^	49	6,3
76. Recruit clinicians with competence in the innovation^b^	1	0,1
*Engage consumers*	31	4,0
41. Involve patients	25	3,2
50. Prepare patients to be active participants	3	0,4
69. Use mass media	3	0,4
*Utilize financial strategies*	2	0,3
1. Access new funding	2	0,3
*Change infrastructure*	9	1,2
11. Change physical structure and equipment	3	0,4
12. Change record system	4	0,5
13. Change service sites	2	0,3

^a^strategy suggested by Boyd et al. [2]. ^b^emerging strategies. Nb. Strategies that could not be linked to activity logs are not represented in this table

*Communication* that was suggested as an additional strategy to ERIC by Boyd et al. [2] was also identified in this study and interpreted as belonging to cluster *5 Train and educate stakeholders*. In addition, four other strategies emerged during data analysis that could not be coded to the ERIC taxonomy or recently suggested additions. These emerging strategies were tentatively named *Recruit clinicians with competence in the innovation*, *Provide stakeholders with time to attend educational meetings*, *Provide stakeholders with resources, and Act as a role model.* Some strategies are described further in conjunction with descriptions of actions and definitions, and all identified strategies with exemplary descriptions and quotes are provided in Additional file 2.

### Actor

A diverse staff was represented as change agents at the health care units. The staff participated in discussions and decisions concerning activities to elicit more PCC in their local setting (see Table 1). Most units reported one or several change agents as the main actors driving different activities to support the implementation of more PCC. Change agents at the DD described how the entire staff at their unit were engaged throughout the organisation to spread information about PCC and used different forums to support buy-in of the concept as well as purporting a PCC leadership. At learning seminars, various actors within and outside the organisation were engaged, serving, for example, as patient representatives, politicians, staff from GPCC, and staff representing other health care units in and outside the region. Some health care units (units 3 and 4) reported actors outside their unit, such as Motivational interviewing-educators, who conducted educational and training activities.

Two health care units (units 1 and 6) deliberately used HCPs within their staff as main actors for part of the change initiative. These HCPs attended university courses to attain a specialist degree in nursing. The student specialist nurses had previously worked as part of the usual staff. They were engaged with planning, enacting, and evaluating the implementation activities they conducted as part of their university degree. The specialist nurses were supported in the change initiative by unit managers and university teachers within the academy, who guided the nurses to use theoretical initiatives (such as SWOT analyses) to tailor their efforts. Moreover, change agents at units 1 and 6 acknowledged these nurses as local opinion leaders or champions at the workplace with good connections with the other HCPs. One change agent at unit 1 described this proficiency in the following terms:

*Then we have XX who is knowledgeable, proficient and driving, and good with the staff. So, XX is really good at persuading staff to come along.* (Dyadic interview, change agent unit 1).

The student specialist nurse at unit 1 worked to promote PCC by attentively listening to and documenting patients’ narratives in the health care record. In unit 6 two student specialist nurses worked with the coordination nurse and other HCPs to introduce a change of the daily round. Unit 5 followed suit in this change initiative. The units’ coordination nurse and the manager were the main actors in introducing the revised daily round for HCPs at this unit.

### Action

In this context the construct action refers to how the strategies were operationalised in a real-world environment, i.e., what activities were enacted within and across the units. Summaries of the actions carried out and number of reported activity logs at the different units are listed in Additional file 4. Results show that discrete educational strategies found in the cluster *Train and educate stakeholders* were used in all units. The DD had a large input in these strategies by arranging learning seminars with up to 200 participants at a time.

*We bring the world to us. Instead of sending out people to take courses…we can reach many by bringing people here instead of sending three people who are then supposed to mediate [PCC].* (Dyadic interview, Change agent at the DD).

Learning seminars involved numerous stakeholders in charge of performing various activities, sometimes altered according to feedback from participating HCPs after each learning seminar. Information and discussions about PCC were delivered through lectures and workshops. Dialogue in workshops enabled protected time for HCPs to discuss the implementation of PCC in their allocated teams. HCPs in learning seminars were given educational materials and informed about the underpinnings of PCC, including its ethical values, the patient law, and the outcomes of PCC. HCPs were encouraged to use PDSA cycles to plan and tailor strategies and evaluate their efforts (Eric 4, 14, 63). Moreover, HCPs’ previous experiences of working to support the implementation of more PCC were shared at the learning seminars. Patient representatives were also involved in sharing their perceptions and health care experiences in discussions about PCC. The learning seminars were registered by change agents at the DD as one activity in the activity log but entailed seven discrete strategies in ERIC (Eric 7, 15, 29, 31, 41, 43, 48). These strategies belong to three clusters in ERIC, of which the most prominent strategy was interpreted as *Conduct educational meetings* (Eric 15)*,* belonging to cluster 5, *Train and educate stakeholders*.

After the learning seminars, change agents at the DD supported those change agents at the health care units who wanted to conduct local educational meetings to reach more staff and create buy-in. The change agents at the health care units were supported with materials, including articles about PCC, power points and suggestions for meeting agendas (Eric 29, 31, 35). Other educational and training strategies operationalised throughout all units were different conversational methodologies, foremost Motivational interviewing [43], and to some extent, Open dialogue [44]. These conversational methodologies contained elements that would aid HCPs to be more person-centred in their approach towards patients.

Four units described how they obtained feedback from patients (Eric 46). Unit 2 regarded a national patient survey as a strategy to gain feedback on their effort while; unit 1 used a “letterbox” at the ward where they prompted patients to give feedback on their care experience; and units 3 and 6 developed surveys to obtain feedback from patients on perceived care.

The strategy *Communication* (new 77) was only identified in two activity logs but discussed in all dyadic interviews and focus groups and seemed to play a major part in everyday implementation efforts. All participants outlined how communication was taking place in various activities, such as discussions between change agents at the units on the phone, in the corridor at the workplace, between colleagues concerning specific patients, at work meetings, and during lunch breaks. Communication of PCC and its implementation was discussed deliberately (in planned agendas) and spontaneously.

Moreover, the emerging strategy *Recruit clinicians with competence in the innovation* (new 76) was reported by units 1 and 2 and based on data from activity logs and dyadic interviews. Change agents delineated how they actively sought to recruit and employ HCPs who had prior experience, competence, and knowledge about PCC. The strategy *Provide stakeholders with the possibility to attend educational meetings* (new 74) is strikingly similar to the ERIC strategy *Conduct educational meetings,* except that units were not always responsible for conducting educational meetings in this context. Instead, they were more inclined to have people participate in meetings to gain more knowledge and skills in PCC without being the driver of the education. All units used this strategy. Early in their implementation, the DD had change agents attending educational activities and conferences throughout the country to learn more about PCC and recommendations for its implementation. In turn, the other health care units enrolled change agents and HCPs in the learning seminars conducted by the DD.

Another strategy that emerged was *Provide stakeholders with resources* (new 75), which resembles *Organize implementation team meetings,* except that it targets individuals given protected time to gain more knowledge, reflect on the innovation, and plan for changes in practice. This strategy is not tied to individuals identified as change agents, early adopters, or other persons with key roles but targeted different HCPs regardless of their function in the implementation process. Lastly, one emerging strategy was identified in interviews but without being linked to any activity log. This strategy was discussed in interviews and was based on change agents portraying themselves as role models. They aspired to meet staff with a person-centred approach to influence them to be more person-centred in their contact with patients. This strategy is tentatively named *Act as a role model (new 78).* One change agent described this strategy as follows:

*I think like this also as a manager. I try to bring this [PCC] with me in my work. If I have a person-centred approach towards staff, it will also affect their meeting with the patient. I mean, how I approach my staff, basically that I make sure that I am person-centred. I hope it will somehow spill over to their attitudes and thoughts about it [PCC].* (Dyadic interview, Change agent Unit 2).

Each unit adapted and operationalised PCC to fit its context and perceived needs to work more in accordance with PCC (Eric 51). Unit 3 chose, for example, to operationalise PCC through increasing patient involvement in treatment. An expanded partnership was proposed by inviting patients to be more active in discussions about the amount of fluid to pull at dialysis and treatment options in home dialysis. Unit 2 chose to increase treatment alternatives for patients to promote more individualised care. A thorough exploration and description of the operationalisation of PCC by each unit will be reported in a separate study.

### Action target

The units mainly reported action targets for whom the strategy targeted. Change agents at the DD reported stakeholders at different hierarchical levels within and outside the organisation as their targets for action. Stakeholders described by the DD as action targets were politicians, managers at different levels within the organisation, union representatives at a national and local level, patient representatives, change agents at the health care units, and change agents at other regions across the country. In contrast, HCPs were most often reported and mentioned as the action target for activities done at the six health care units. Strategies targeting patients and their next of kin with information about the change towards more PCC at each unit and across the region (Eric 50, 69) were scarce (*n* = 6).

Reports on strategies targeting conceptual targets (e.g., attitudes, skills, knowledge) were found mainly concerning increased knowledge. Increased knowledge and understanding of PCC through different educational activities were described by all units as a conceptual target. Moreover, several units used different strategies to enable cooperation, understanding, and a common goal to strive for between HCPs with various vocational roles within teams or between other units (the DD). Strategies aimed at affecting HCPs conversational skills were also mentioned as a conceptual target for those units that reported training in Motivational interviewing. Another conceptual target that the DD and unit 4 reported was any change targeting the electronic health care records by adding the search word “narrative” and documentation of health care plans.

### Temporality

**Fig. 3:**
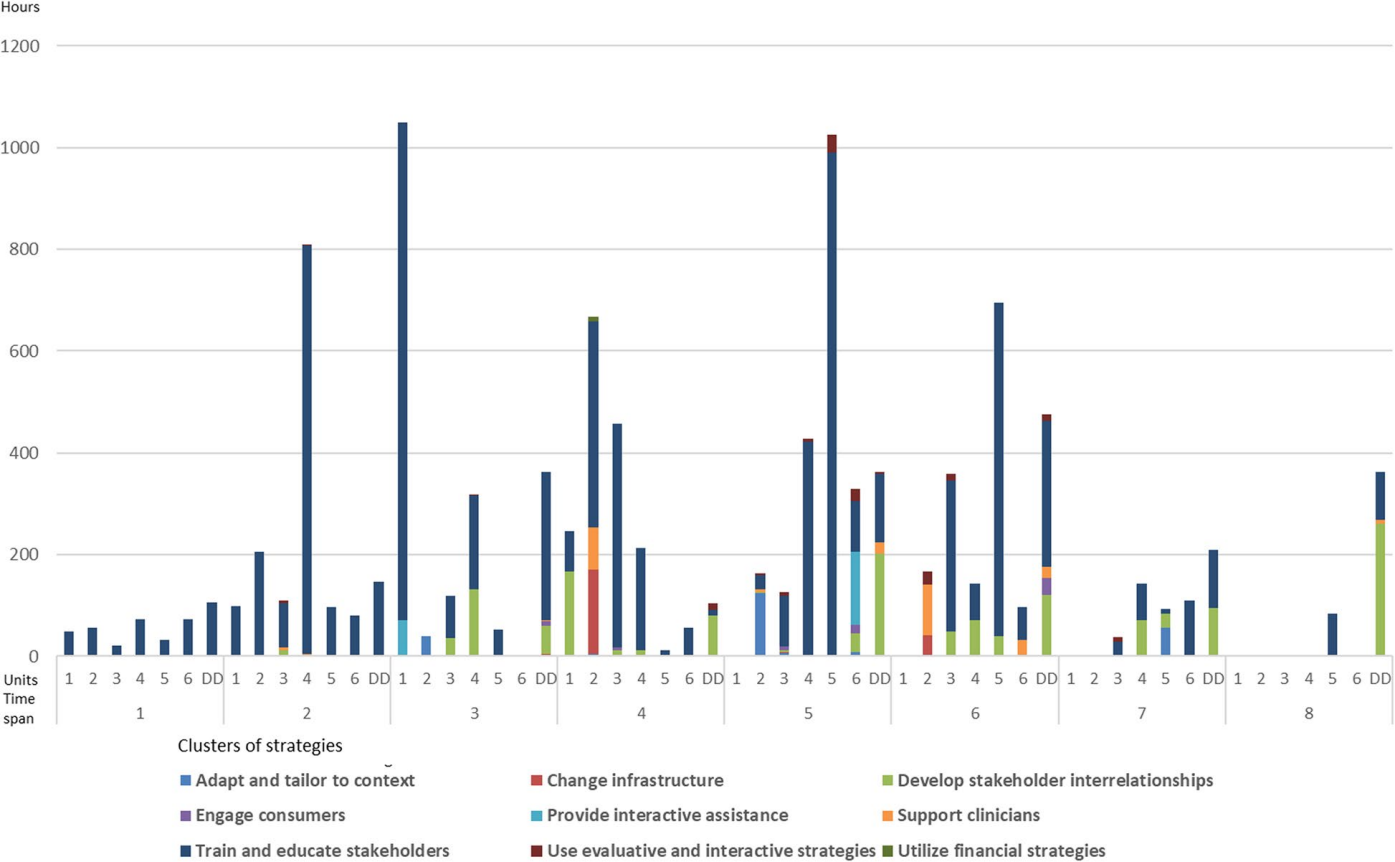
Strategies summed in ERIC clusters and dose on a half-year time span at each unit from May 2016 until November 2019. Nb. time spans 1 and 2 represent retrospective data collection while time spans 3 to 8 represent prospective data collection

All embedded units primarily reported educational activities in the first two periods. These activities were coded to strategies in the ERIC cluster *train and educate stakeholders* (see Fig. 3 for clustered strategies in all units by time). Such activities included conducting or participating in learning seminars, which many of the units saw as the starting point for their work to transition to more PCC. After the first round of learning seminars, all units brought their newfound knowledge and information back to their workplace and arranged educational meetings for HCPs at their unit. Unit 3 continued to enrol HCPs for learning seminars each year until the last two periods, which becomes evident in strategy frequencies showing that unit 3 reported 49% of all the logs found in the emerging strategy *Provide stakeholders with time to attend educational meetings.* Units 2 and 4 had the same intention but had to abandon this plan due to shortness of staff. For the same reason, unit 1 had no reports in the activity log from period 5 and onwards and seemed to rely on HCPs to build on what some HCPs already had learned and then learn from one another in daily work. One change agent described this approach as:

*I think we’ve just let it catch on if I can put it that way, so we haven’t shown and asked people to watch, do this, or do that. We let it take its time.*

*Hopefully, they will discover what is good, and then it will just catch on.* (Dyadic interview, Change agent unit 1).

Likewise, units 5 and 6 were merged due to shortness of staff after taking part in the first learning seminar in May 2016. Therefore, change agents at these units put their work to implement more PCC on partial hold during periods 3 and 4, hoping that the units would be subsequently separated. In the end, they stayed merged and decided to start their work to implement more PCC regardless of their organisational situation.

Reported activities other than those belonging to the cluster *Train and educate stakeholders* increased from period 3 onward. Activities connected to remodelling the ward at unit 2 belonging to the cluster *Change infrastructure*, were reported in periods 4 and 6. The DD continued to support the health care units in the region throughout the timespan by inviting units to its learning seminars and other educational events, as well as working to build interrelationships within and outside the region.

All involved units discussed in interviews how implementing PCC was expected to take time and effort, often saying, “this is going to take time and we just need to keep it up”.

A change agent from unit 2 elaborated on this issue by saying:

*In other words, it’s going to, and it’s ok to take small steps. It’s ok to have setbacks, and it’s ok…because we have a really long plan. That’s where we’re heading. And we need to keep it up all the way. This is nothing temporary. It’s something that we are going to work with for a long time.* (Dyadic interview, change agent unit 2).

### Dose

**Fig. 4:**
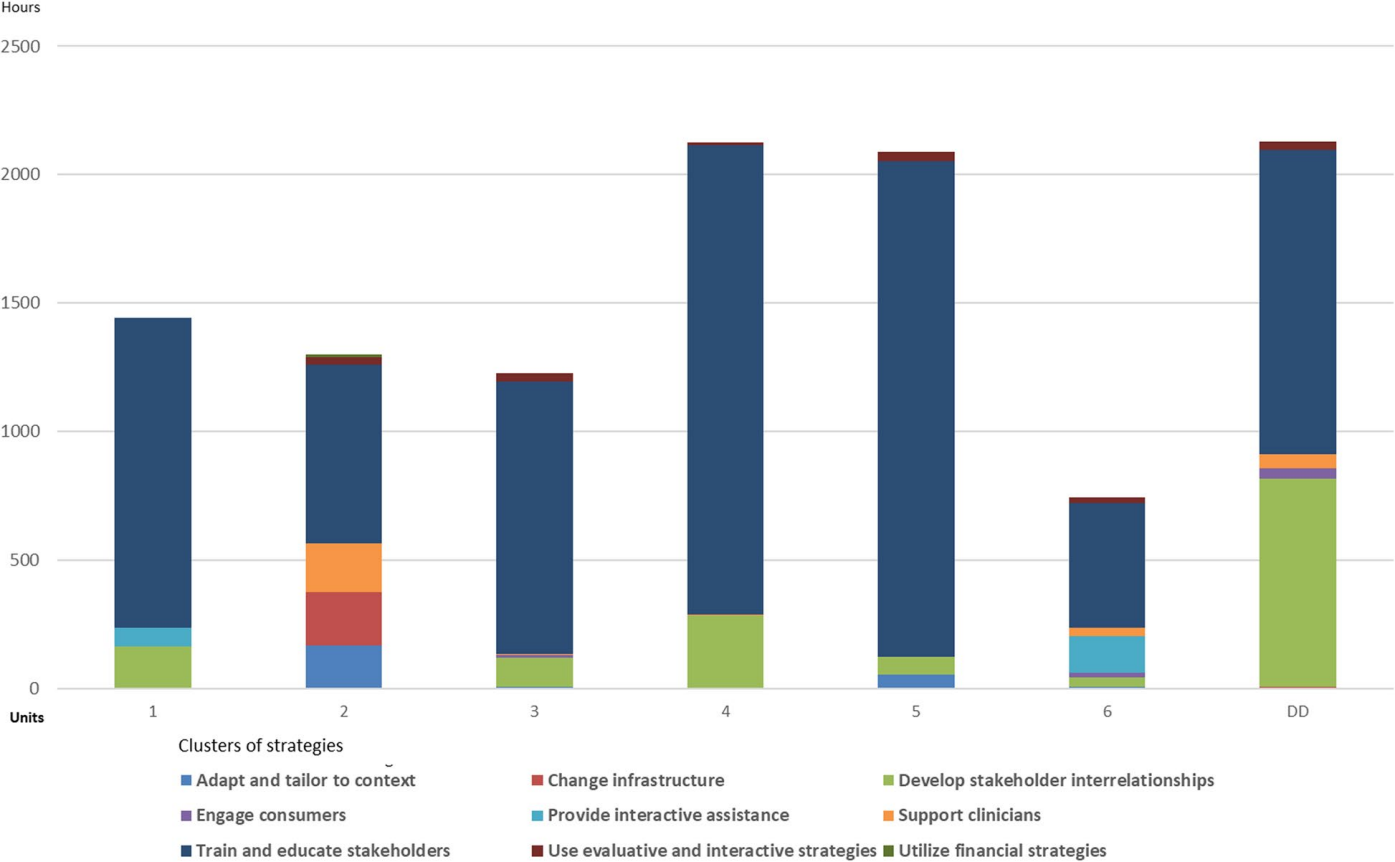
Activity logs summed in ERIC clusters and total dose across all embedded units

From 413 reported activities, the frequency of activities directly targeted towards HCPs engaged in adopting and integrating PCC in their practice was 159 (38,5%) compared to 254 (61.5%) activities targeting change agents and stakeholders in charge of supporting the implementation initiative. The activities generated a total dose of 11,076 person-hours across all units, of which 9028 (81.5%) person-hours were spent on HCPs delivering PCC and 2048 (18.5%) on strategies targeting support functions. Figure 4 displays each unit’s dose in person-hours to ERIC clusters.

### Implementation outcome affected

Descriptions of intended outcomes for enacted discrete strategies were for several reasons challenging to identify in all units. Reports on outcomes in logs were made for an activity that, as previously mentioned, could contain more than one discrete strategy and thereby making it impossible to separate specific outcomes for each strategy. Likewise, when change agents discussed the chosen strategies for implementation outcome during the interviews, these discussions were often based on enacted activities. A notion expressed by all change agents was the overarching implementation goal to increase the quality of care for patients by working more consistently with providing PCC. More specifically, some change agents described how various education and training activities aimed to reach all HCPs, leading to patients receiving equal care regardless of who they encountered at their unit. Change agents at unit 5 described how daily discussions and training for HCPs (Eric 19) aimed to affect HCPs acceptance of PCC and its operationalisation at the ward. Unit 3 used consensus meetings for all HCPs to cast votes on operationalising PCC and which activities should be prioritised. The outcome for this strategy was to devise a plan and set up goals for the implementation effort, create buy-in for HCP, and motivate an acceptance for the change initiative. Change agents at all units described how being observed through a research initiative was a strategy to evaluate their efforts and outcomes, characterised by a change agent from unit 1 as:

*The advantage of having you come in is that it will be neutral. I can imagine that there will be patients who may think the approach wasn’t what I had wished for. Then I think it’s an advantage that it [the evaluation] comes from someone neutral [the research group].* (Dyadic interview, change agent at unit 1).

Descriptions of perceived challenges about evaluating the efforts were common and described by a change agent from unit 2 as:

*We have also talked about what kind of measures we should use. We haven’t found anything right yet. It’s an ongoing discussion.* (Dyadic interview, Change agent at unit 2).

### Justification

Change agents at the DD described how theoretical frameworks for dissemination and implementation guided some of their chosen implementation strategies. Change agents at the DD had previous knowledge of leadership theories. They had also received information about implementation of PCC by participating in national conferences about PCC, where its implementation was discussed. These information sources and knowledge guided some of their justifications for selected strategies. Chosen strategies were also inspired by the theoretical underpinnings of the innovation itself, i.e., PCC. Change agents at the DD described how their approach was consistent with teachings from PCC, where they regarded staff at each health care unit through a person-centred lens. This approach meant that change agents and their colleagues were viewed as resourceful and autonomous experts in their field and capable of choosing strategies based on their individual and unique knowledge and experiences of their local context, albeit with support from the DD (Eric 63). One of the change agents at the DD described it in the following way:

*Our strategy is to be person-centred in our support; you know, listening to their narratives. What are your thoughts on this? What do you think is difficult? What’s the next step? Together with the managers, we can discuss and produce something that feels ok. We need to be person-centred, as XX says.*

*Yes, we have our expert knowledge within quality and improvement work, but they have expert knowledge from the way it is out there. So, we need to agree on, well, something that’s reasonable.* (Dyadic interview, Change agent at the DD).

In contrast, change agents at the health care units seldom described or reported any theoretical justification for their strategies. Rather, they based their strategies on local knowledge from their work context, including staff, patient characteristics, experience from earlier change efforts, and pragmatic factors such as resources.

## Discussion

Our study reveals how implementation strategies were enacted over time to support implementation of PCC in a real-world setting. Change agents and HCPs in 7 units used several strategies to support their change initiative.

### Common strategies

We identified 43 discrete strategies in this study. Our result is consistent with similar research that identified 36 to 45 discrete strategies [7, 9, 10]. These 43 discrete strategies represented all clusters in ERIC, although the use of the strategies varied widely (frequency from 1 to 110). The highest reported frequencies were found in the cluster *Train and educate stakeholders* (40.3%) and *Develop stakeholder interrelationships* (38%). It is common in implementation initiatives to include educational activities for those who are supposed to deliver the innovation [7, 45]. Such activities have previously rendered small to moderate changes in HCPs practice [13]. However, up to eight discrete strategies serving different learning styles were identified in learning seminars, educational meetings, and training sessions, in this study. The use of an approach consisting of a mix of interactive and learning activities is supported by previous studies [13, 46] and given high importance and feasibility ratings from experts in the field of implementation [40]. The intense focus on educational activities in the studied implementation initiative is probably because the overall support strategy developed by the DD contained many such strategies, which is then reflected in the strategies chosen at all health care units. This comprehensive strategy might have inspired the change agents at the health care units, who had limited knowledge and experience of leading implementation, to continue with similar activities at their units.

### Less common strategies

Discrete strategies belonging to the cluster *Use evaluative and iterative strategies* were scarce in this study (4.6%), suggesting a possible knowledge gap in implementation efforts in a real-world setting. Strategies within this cluster have received high importance and feasibility ratings from experts in implementation science and clinical practice [40], indicating a possible lack of these crucial strategies in the studied implementation efforts. Change agents at the DD encouraged the HCPs who participated in learning seminars to use PDSA cycles to regularly monitor and evaluate their implementation efforts [47]. However, reports on evaluations in the activity logs were rare. Furthermore, discussions in interviews and focus groups with change agents at the health care units were primarily focused on the “final” outcome, i.e., patients’ perception of PCC rather than tracking process outcomes such as fidelity and acceptability [48]. In comparison, despite a lack of reports in the activity logs, change agents at the DD described how they used feedback from participating HCPs after each learning seminar to evaluate their strategies regularly. If enacted strategies are not evaluated over time, change agents might be unaware of the outcome and thus miss the opportunity to change the implementation support if needed. The limited use of evaluative strategies may be due to the units opting to be part of the research initiative. Some change agents described how the outcome of the implementation initiative was challenging to grasp and being part of a research initiative would give them much-needed feedback on the work they performed.

### Tailoring

The ERIC strategy *tailoring* (Eric 63) appeared in this study to rely on change agents’ intuition and pragmatic discussions based on experience. We could only identify one report in the activity logs from the health care units, indicating that a formal evaluation had taken place to guide any tailored strategies. However, we did identify this strategy in some of the interviews. Change agents at the DD tailored their implementation strategies based on literature, knowledge gained at conferences and shared experiences of implementation of PCC by change agents from other regions. Feedback from HCPs participating in learning seminars was also evaluated and used as a base to adjust the chosen strategies. This result is different from those observed by Bunger et al., where tailoring strategies were reported as the most common enacted strategy [7]. Change agents in the current study had the invaluable experience of the context for implementation and justified their chosen strategies based on these experiences and intuition. However, they seemed to lack experience in conducting systematic assessments in which strategies were chosen deliberately and to address the most acute and pertinent barriers to implementation. While most theories and frameworks in implementation science advocate using different methods to select and link strategies to identified barriers, so far, research supporting this approach is still in its early stages [49]. To match strategies with identified determinants has shown inconsistent results and has proven to be a complex task for researchers in implementation science [12].

### Involvement of patients

Patients are a prerequisite and a core aspect of PCC [15] and are often considered an essential determinant in implementation models and frameworks [44]. We were surprised to find few reports or descriptions from units on strategies directed to inform and prepare patients about the change initiative (Eric 50). In one activity log change agents at the DD reported using mass media to disseminate information to all inhabitants in the region. Other initiatives to disburse information seemed to be directed primarily towards actors (e.g., HCPs, politicians, and managers) at different organisational levels. Reaching out to the inhabitants in the region could perhaps aid implementation by creating increased demand and acceptance by the public to be active partners in care. Informing patients about PCC is limited in today’s implementation efforts in Sweden according to a recently published nationwide report [50]. Increased and upgraded information about patients’ rights in health care is strongly recommended by the Swedish agency for health and care services analysis [50].

### Involvement of students as actors

Another elaborate strategy reported in activity logs from units 1 and 6 was the implementation activities conducted by nurses enrolled in a specialist degree at the university. They had substantial input as actors at these units. The strategy of using the support from HCPs to train their colleagues in the PCC approach at the wards was coded as *Provide clinical supervision* (Eric 53). Clinical supervision provided by peers in education can be regarded as a beneficial solution for managers and HCPs enrolled in specialist degrees in similar contexts. The three student specialist nurses were regarded as local opinion leaders or champions by the change agents. The use of local opinion leaders or champions has been identified and recommended as a promising strategy to increase efficacy in implementation efforts [51–53]. HCPs in higher specialist education who, as part of their training, take on a role as an actor in implementation initiatives, might be a strategy that stakeholders in a real-world setting should consider more often.

### Methodological considerations

In our analysis we used a codebook with names, definitions, and clusters from ERIC [5]. We also used inclusion and exclusion criteria for each strategy, which was an important asset to help identify the relative frequency of discrete strategies. However, we found that using ERIC together with recommendations for specifying strategies in line with Proctor [3] to identify, name, and define strategies were in some cases overlapping and a complex endeavour in this natural setting. As Proctor et al. [3] advocate a need to define actor and action target in their recommendations, it is not necessary to have these specifications built into some of the strategy names found in ERIC (e.g., champion and local opinion leader). Moreover, some of the clusters in ERIC [40] are clearly directed towards the action target (e.g., *Train and educate stakeholders* or *Support clinicians)*, making the coding even more challenging. Our observations are in line with other studies that have highlighted some discrepancies concerning the level of detail across different ERIC strategies [7, 54, 55]. We also acknowledge that other ways of clustering ERIC strategies have been applied in other research initiatives to suit different study objectives [2, 7, 8, 10, 54].

The challenges of coding in our study were further transferred to the specification of dose associated with each strategy. Because dose was quantified based on person-hours invested in the reported activities, it was impossible to sort out a dose for each strategy identified in activities containing more than one strategy. Moreover, in those cases where one activity in the log included more than one strategy located over several clusters, the dose was reported to the cluster with the strategy identified as most pertinent for each activity. Thus, strategies represented by other clusters became indiscernible in relation to dose in the results section. Identifying several strategies within one activity is common in similar research [10], as well as facing challenges related to quantifying dose for each strategy [56]. Despite these challenges we still believe that it is important for researchers and stakeholders to report both the frequencies of strategies, and their dose. In our study a frequency of more than 60% of the strategies were directed towards support functions while the dose of those strategies (i.e., person-hours) summed up to less than 20%. This gives us insights that strategies may differ in e.g., their target and actions but also regarding the dose used and required to carry out a strategy. Thus, some strategies may be pertinent for effective implementation but may not require a lot of resources and vice versa. Such knowledge is of great value for stakeholders who are charged with meeting budget demands and still want to succeed with their implementation efforts. With future studies we hope that the knowledge on strategy specifications, including dose, will lead to increased understanding of the strategies used in implementation.

Some discrete strategies, such as *Act as a role model* and *Communication,* may be difficult to report regarding dose also in future studies. Nonetheless, we believe that these strategies are important to report as they have been identified as implementation strategies and may prove pertinent to implementation success. *Communication* has been identified in other studies and is probably a strategy that can be generalised across most implementation efforts and should perhaps be added to ERIC [2, 7]. On the other hand, *Act as a role model* may be more tied to specific innovation characteristics such as those identified in PCC [20] and have been identified as a strategy used by managers in another PCC study [57]. Our observations are similar to those of other researchers describing perceived challenges to capture data representing activities of a more informal character (e.g., discussions in the corridor or at lunch breaks) [2, 7].

Several factors may limit the results of this study. First, logbooks were based on self-reported information from change agents at the units. Reports on activities enacted from the first year were based on recall and consultation of their calendars and other documents. Results from the first year’s activity logs were almost exclusively based on different formal educational activities instead of activities of a more informal character, such as discussions on promoting *adaptability* (Eric 51). Even though it is common in implementation work to begin with educational activities, it is possible that results from activity logs in the first-year data underestimate some of the work carried out at the different units.

Because logbooks were based on self-reports, we do not know if some change agents were more consistent and detailed in their reports than others. Self-reports could have created an inaccurate description of the case and the comparison between units. However, logbooks were triangulated with data from documents, dyadic interviews and focus groups to validate data and increase our understanding of the enacted strategies. Triangulating data was an important aspect of this study as data from activity logs sometimes lacked detail or were missing. Thus, we acknowledge that the data sources contributed to varying degrees in detailing the different strategy characteristics. Activity logs were e.g., invaluable for identifying actors, action target (the person(s) targeted by the strategy), temporality, and dose while interviews gave a deeper understanding of particularly conceptual targets, justification and for identifying multiple discrete strategies in a single activity log. Moreover, some strategies that emerged from the interviews could not be linked to any activity logs and interviews indicated that some strategies identified in logs were underrepresented (e.g., *Communication)*. In addition, despite our efforts, we likely missed data on formal and informal activities conducted at the health care units.

Lastly, the effectiveness and HCPs perceptions of the strategies reported in this study have not been evaluated thus far, inhibiting discussion of the implementation initiative in connection with the outcome as a whole or each strategy’s relative contribution towards a possible outcome.

## Conclusion

This study represents an example of implementation of PCC in a real-world setting without support from researchers. We triangulated data sources to illustrate which strategies were chosen and enacted to support implementation of PCC in 7 units in a health care region. The study results can serve as a valuable contribution to the research field on future implementation initiatives of PCC and further guidance in the methodology for tracking implementation strategies in a real-world setting.

We propose that strategies chosen based on experience and intuition from a real-world setting may be supplemented with implementation theories, frameworks, and guidelines in future work to build knowledge on the large-scale implementation of PCC. Moreover, the noticeable dominance of identified strategies belonging to the two ERIC clusters - *Develop stakeholder interrelationships* and *Train and educate stakeholders* - lead us to believe that it may be of value to consider increased use of other strategies in future implementation efforts. Strategies such as those targeting evaluations and measures of implementation outcome along with strategies focusing on informing patients about the implementation effort could be considered. To increase and build on the knowledge and experience that change agents and HCPs have within the context of an organisation, we also advocate for increased collaboration between implementation scientists and practitioners in future PCC implementation efforts.

## Supplementary Information

**Additional file 1.** Interview guide

**Additional file 2.** Coding manual based on ERIC number, names and definitions including inclusion and exclusion criteria, and examples of data sources.

**Additional file 3.** Example of discrete strategies named by ERIC and specified using Proctor et al.’s recommendations.

**Additional file 4.** Summaries of enacted activities at the embedded units.

## Data Availability

The datasets used in this study are available from the corresponding author on reasonable request.

## Abbreviations

ERIC: The Expert Recommendations for Implementing Change
HCP: Health care professional
PCC: Person-centred care
GPCC: The Gothenburg University Centre for Person-Centred Care
