# Supplementary material for: Tracking, naming, specifying, and comparing implementation strategies for person-centred care in a real-world setting: a case study with seven embedded units
Source: BMC Health Serv Res. 2022 Nov 24;22:1409. doi: 10.1186/s12913-022-08846-x (PMC9685853; doi:10.1186/s12913-022-08846-x)
Supplement: Supplementary file 2 — Additional file 2. Coding manual based on ERIC number, names and definitions including inclusion and exclusion criteria, and examples of data sources. [file 12913_2022_8846_MOESM2_ESM.docx]

**Additional file 2.**

| **Strategy number, name, and definition* [1, 2]** | **Inclusion and exclusion criteria based on the innovation and its context** | **Example(s) and data source** |
| --- | --- | --- |
| 1. **Use evaluative and iterative strategies** | | |
| **4. Assess for readiness and identify barriers and facilitators**  Assess various aspects of an organization to determine its degree of readiness to implement, barriers that may impede implementation, and strengths that can be used in the implementation effort. | **Inclusion criteria:** Include statements indicating that a strategy focusing on a formal process to assess readiness or identifying barriers and facilitators have been conducted.  **Exclusion criteria:** Exclude statements where it is unclear if this activity has been conducted (e.g., informal statements about perceived determinants of practice). | Project group 1: all participants are active and write notes when we do a SWOT analysis. The aim is to find barriers that we need to prevent.  Log unit 6. |
| **5. Audit and provide feedback**  Collect and summarize clinical performance data over a specified time period and give it to clinicians and administrators to monitor, evaluate, and modify provider behavior. | **Inclusion criteria:** Include statements indicating that clinical performance data have been collected and summarised and then provided as feedback anyhow to stakeholders and HCPs.  **Exclusion criteria:** Exclude statements indicating that feedback has been provided but that an audit has not been based on a formal process. Code statements of feedback without an audit to 77. Communication or 58. Remind clinicians. | Information about the unit’s strategic plan and strategic goals. Feedback [to HCPs] on the results from the national quality register and patients’ perception of care at the unit. Logg unit 2. |
| **14. Conduct cyclical small tests of change**  Implement changes in a cyclical fashion using small tests of change before taking changes system-wide. Tests of change benefit from systematic measurement, and results of the tests of change are studied for insights on how to do better. This process continues serially over time, and refinement is added with each cycle. | **Inclusion criteria:** Include statements indicating that changes have purposely started on a small scale with the intent to assess PCC in parts with different patient groups, contexts, or vocational roles. Include statements based on discussions among those involved in the work, even if it is not based on systematic measurement.  **Exclusion criteria:** Exclude statements indicating that it is unclear that the intent was to start on a small scale before scaling up. | Discussion and test of bedside reports. We attempted to do the rounds in this way for a period but then decided to finish the trial for now. Logg unit 6.  Yes, but we chose to start on a small scale [HCPs talk about, listening to patients’ narrative and its documentation, tested in one of the multibed rooms of the ward] and then built on across the whole unit. Dyadic interview unit 1. |
| **23. Develop a formal implementation blueprint**  Develop a formal implementation blueprint that includes all goals and strategies. The blueprint should include: 1) aim/purpose of the implementation; 2) scope of the change (e.g., what organizational units are affected); 3) timeframe and milestones; and 4) appropriate performance/progress measures. Use and update this plan to guide the implementation effort over time. | **Inclusion criteria:** Include statements indicating that a plan for implementation of PCC has been developed and/or used/updated to guide the implementation effort over time.  **Exclusion criteria:** Exclude statements related to planning and recording of specific activities, meeting protocols and such and code accordingly. e.g., 56. Purposely re-examine the implementation. | Creation and rewriting of an action plan for PCC for planning.  Logg unit 4.  We have made an action plan and continue to work in smaller groups where staff are more involved overall.  Focusgroup unit 3. |
| **46. Obtain and use patients/consumers and family feedback**  Develop strategies to increase patient/consumer and family feedback on the implementation effort. | **Inclusion criteria:** Include statements indicating feedback based on surveys, complaint forms, improvement suggestion forms, verbal feedback through formal or informal narratives from patients/next of kin if discussed jointly at the unit or with the department for development.  **Exclusion criteria:** Exclude statements related to feedback from stakeholders and change agents and code to 5. Audit and provide feedback. | We get feedback on the results from the national patient survey to raise awareness of patients views on the care. Dyadic interview unit 2.  We have a letterbox at the ward. Every patient who is on their way to leave gets a little survey. What are your perceptions, is it something that you would like improved and so? Far from everybody turns it in, but some do. And then we get feedback on their perceptions. Dyadic interview unit 1. |
| **56. Purposely reexamine the implementation**  Monitor progress and adjust clinical practices and implementation strategies to continuously improve the quality of care.  determining when adjustments are needed have also been found to be useful. | **Inclusion criteria:** Include statements related to implementation activities that have been conducted and monitored and where feedback is provided to stakeholders and HCPs on their efforts.  **Exclusion criteria:** Exclude statements related to working directly with the implementation plan, i.e., updating and code to 23. Develop a formal implementation blueprint. | Continued compilation from the planning days and other work with PCC… overview of what has been done and what we will do. Log unit 4.  Evaluation of the project [implementation of PCC team round] and what we need to review to improve. Log unit 5. |
| 1. **Provide interactive assistance** | | |
| **53. Provide clinical supervision**  Provide clinicians with ongoing supervision focusing on the innovation. Provide training for clinical supervisors who will supervise clinicians who provide the innovation. | **Inclusion criteria:** Include statements indicating that supervision has been performed in practice settings. Training for clinical supervisors does not have to be conducted by change agents but can be undertaken in mutual understanding or collaboration with other stakeholders such as academic institutions in charge of the training/education as part of extra curriculum courses as part of HCPs specialist degrees.  **Exclusion criteria:** In cases where supervision is conducted regularly but without making a clear definition of the supervisor role is coded to 19. Conduct ongoing training. Exclude statements related to activities that involve discussions between clinicians with no apparent supervisor and code to, e.g., 48. Organise clinician implementation team meetings. | Meeting in the local PCC group. To Support collegial supervision for the implementation of PCC. Log unit 4.  Start-up of the new daily round. A new day structure and new round templates are used. XX and XX are at the unit to support and inspire the staff up until [date]. Log unit 6. |
| 1. **Adapt and tailor to context** | | |
| **51. Promote adaptability**  Identify the ways a clinical innovation can be tailored to meet local needs and clarify which elements of the innovation must be maintained to preserve fidelity. | **Inclusion criteria:** Include statements indicating that PCC has been adapted in a conscious process to fit patient needs and local context regardless of elements of fidelity.  **Exclusion criteria:** Exclude statements where PCC has been adapted but without a conscious and clear notion about this. Exclude statements indicating that, e.g., evaluative and iterative strategies such as 4. Assess for readiness and identify barriers and facilitators have been conducted without suggesting that it has been followed with justified adaptations.  Authors comments: research points to PCC as a highly complex concept with multiple meanings, definitions and operationalisations throughout research and practice making discussions about fidelity uncertain. | To individualise rehabilitation for neurology patients, e.g., stroke and MS start horse support therapy. This gives us another form of treatment to offer when we discuss what is best for the patients and their wishes regarding rehabilitation. Log unit 2. |
| **63. Tailor strategies**  Tailor the implementation strategies to address barriers and leverage facilitators that were identified through earlier data collection. | **Inclusion criteria:** Include statements indicating that strategies to implement the innovation have been selected to match barriers and facilitators identified by a formal and conscious process.  **Exclusion criteria:** Exclude statements indicating that strategies have been modified without support from earlier data collection or a formal and conscious process. | [The aim is to find barriers that we need to prevent] The coordination nurse suggests for a changed day schedule. Log unit 6 |
| 1. **Develop stakeholder relationships** | | |
| **6. Build a coalition**  Recruit and cultivate relationships with partners in the implementation effort. | **Inclusion criteria:** Include statements indicating that implementation efforts are conducted in partnership with other stakeholders. Use this code even if a coalition member has not been the instigator of the coalition if the member continues building relationships in the implementation effort. Some stakeholders are not actively building or initiating the alliance but acting as involved and active partners in the coalition, sometimes driving the continuing relationships.  **Exclusion criteria:** Exclude statements indicating that a group of stakeholders are part of an existing structure and code to 40. Involve executive boards. Exclude statements indicating that a group has been formed to give advice and input on the implementation effort and code to 64. Use advisory boards and workgroups. | Central group for creating support in *Take care* [electronic health care record] to document PCC. Representatives for the central part of the workaround implementation of PCC [the DD] and representatives from XX in XX and XX.  Log unit 4. |
| **7. Capture and share local knowledge**  Capture local knowledge from implementation sites on how implementers and clinicians made something work in their setting and then share it with other sites. | **Inclusion criteria:** Include all statements where the focus is to share local knowledge from different settings, to other sites (through for example educational meetings or educational material).  **Exclusion criteria:** Exclude statements where stakeholders are, e.g., involved to talk about PCC but without giving any reference to own or local experience of working with PCC and code to, e.g., 15. Conduct educational meetings. | You know, we work with them by pulling. It’s not like that we push out our support just like that, but for example, XX and XX had their teams come and talk at the follow-up seminar, and now we have had the chance to invite them, from XX unit to a major seminar in November in Stockholm, [to talk] about PCC. Dyadic interview unit DD. |
| **17. Conduct local consensus discussions**  Include local providers and other stakeholders in discussions that address whether the chosen problem is important and whether the clinical innovation to address it is appropriate. | **Inclusion criteria:** Include statements indicating that discussions are conducted in mutual respect and understanding between the people driving the change towards more PCC and other stakeholders such as clinicians in the organisation.  **Exclusion criteria:** Exclude statements indicating that, e.g., meetings were not focused on consensus discussions but information sharing and code to, e.g., 15. Conduct educational meetings. | Yes, we have our expert knowledge within quality and improvement work, but they have expert knowledge from the way it is out there. So, we need to agree with what is reasonable. Dyadic interview unit DD. |
| **24. Develop academic partnerships**  Partner with a university or academic unit for the purposes of shared training and bringing research skills to an implementation project. | **Inclusion criteria:** Include statements indicating that academic partnership was developed for shared training or evaluation purposes related directly to the implementation project.  **Exclusion criteria:** Exclude statements indicating that stakeholders and HCPs have taken part in extra-academic curriculum courses or conferences to learn more about PCC or implementation if the purpose is not shared training or other cooperation-related directly to the implementation project. | When we started to discuss this here at the ward office that you had invited us, we had to think about whether this was an alternative. And then when we had made the decision, we said, we have nothing to lose. Basically, it’s another form of support.  Dyadic interview unit 1. |
| **35.Identify and prepare champions**  Identify and prepare individuals who dedicate themselves to supporting, marketing, and driving through an implementation, overcoming indifference or resistance that the intervention may provoke in an organization. | **Inclusion criteria:** Include statements indicating that individuals have been identified and prepared to support more PCC changes.  **Exclusion criteria:** Exclude statements indicating that HCPs have been organised in teams to support one another and code instead to 48. Organize clinician implementation team meetings. | And then we decided that it was management who should participate in this training together with key people. We should find a few people who were…had that sort of role in the unit [champion, local opinion leader] so that the patients receive maximal benefit from those people and that we spread it [PCC] throughout the unit.  We also thought it was important to have a mix of professions [taking part in seminars].  Focus group unit 4. |
| **40. Involve executive boards**  Involve existing governing structures (e.g., boards of directors, medical staff boards of governance) in the implementation effort, including the review of data on implementation processes. | **Inclusion criteria:** Include statements related to existing groups that support the implementation of PCC.  **Exclusion criteria:** Exclude statements where it is not stated that an existing group makes up the board. Code instead to 6. Build a coalition or 64. Use advisory boards and workgroups. | Education day. Region Dalarna, Psychiatry, Politics. Continued support to work with PCC.  Log unit DD.  Health and health care director responsible for the meeting, members in the health care council, 1^st^ line managers, and Administration. Support from DD who conduct recurrent meetings for nurses with health care-specific themes that also touch on PCC.  Log unit DD. |
| **48. Organize clinician implementation team meetings**  Develop and support teams of clinicians who are implementing the innovation and give them protected time to reflect on the implementation effort, share lessons learned, and support one another’s learning. | **Inclusion criteria:** Include statements indicating that clinicians have been involved in team activities where implementation of the innovation is discussed.  **Exclusion criteria:** Exclude statements indicating that clinicians have been provided with resources, i.e., protected time to learn more about the innovation but not through team activities. Code instead to 75. Provide clinicians with resources. | Health care personnel – team meeting  Log unit 3. |
| **52. Promote network weaving**  Identify and build on existing high quality working relationships and networks within and outside the organization, organizational units, teams, etc. to promote information sharing, collaborative problem-solving, and a shared vision/goal related to implementing the innovation. | **Inclusion criteria:** Include statements indicating cooperation of many diverse activities to implement more PCC.  **Exclusion criteria:** Exclude statements where cooperation is not the focus of the activity (e.g., going to a conference to listen to stakeholders talking about PCC). | National cooperation and network [meeting] around standardisation [PCC] at SALAR  Log unit DD. |
| **57. Recruit, designate, and train for leadership**  Recruit, designate, and train leaders for the change effort. | **Inclusion criteria:** Include statements indicating that leaders have been trained for the specific change effort.  **Exclusion criteria:** Exclude statements indicating that leaders have not been subject to any activities targeting training or increased knowledge in the change effort. | XX participated in the healthcare associations leadership training course where one of the course books was PCC in line with GPCC.  Log unit 5. |
| **64. Use advisory boards and workgroups**  Create and engage a formal group of multiple kinds of stakeholders to provide input and advice on implementation efforts and to elicit recommendations for improvements. | **Inclusion criteria:** Include all statements indicating that different stakeholders are engaged in a group to provide input on the specific parts or in the total implementation effort.  **Exclusion criteria:** Exclude statements indicating that the group already has other main priorities or roles and code to, e.g., 40. Involve executive boards or 24. Develop academic partnerships. | Reference group meeting with interprofessional support staff in quick chat to integrate and develop e-health services to support more PCC.  Log unit DD. |
| **65. Use an implementation advisor**  Seek guidance from experts in implementation. | **Inclusion criteria:** Include statements where guidance have been sought from experts on the implementation initiative.  **Exclusion criteria:** Exclude statements where information and experiences have been shared between stakeholders and code instead to, e.g., 6. Build a coalition, or 7. Capture and share local knowledge. | Discussion and advice for continuation [from change agents at the DD]  Log unit 4.  They [change agents from the DD] participated in a meeting here with us. They thought we should do this on our own.  We described what we thought or how we thought, and we wanted confirmation from them that we were on the right track.  And they gave us some ideas about how you could…raise certain things, or what activities you could do, and so on.  Focus group unit 3. |
| 1. **Train and educate stakeholders** | | |
| **15. Conduct educational meetings**  Hold meetings targeted toward different stakeholder groups (e.g., providers, administrators, other organizational stakeholders, and community, patient/consumer, and family stakeholders) to teach them about the clinical innovation. | **Inclusion criteria:** Include statements indicating that the unit “itself” has developed and staged the educational meeting.  **Exclusion criteria:** Exclude statements indicating that stakeholders and HCPs have taken part in educational meetings, but the unit they belong to had no role in planning or staging the meeting. Code instead to 74. Participate in educational meetings | Yes, I mean, the reasoning behind these learning seminars from the beginning, and I think it’s still the case. We have created places and platforms for teams, where you have the chance to both gain knowledge in terms of research and see what others have done. It’s also connected to e-health. But it’s still the teams themselves who need to apply, who need to be interested and come along. We can only, like, offer a place and say that this exists, it’s free, and you’re welcome to apply. But the drive and the work have always come from the managers and the teams themselves. And we stand for that strategy.  Dyadic interview unit DD. |
| ***74. Provide stakeholders with time to attend educational meetings** | Provide stakeholders and HCPs with the possibility to attend educational meetings targeted toward different stakeholder groups (e.g., providers, administrators, other organisational stakeholders, and community, patient/consumer, and family stakeholders) to learn about the innovation.  **Inclusion criteria:** Include statements for which stakeholders and HCPs have participated in educational meetings planned and conducted by others.  **Exclusion criteria:** Exclude statements where the educational meetings have been orchestrated by their unit and code instead to 15. Conduct educational meetings. | Management education, including elements of PCC  Log unit 5. |
| **16. Conduct educational outreach visits**  Have a trained person meet with providers in their practice settings to educate providers about the clinical innovation with the intent of changing the provider’s practice. | **Inclusion criteria:** Include statements related to trained persons visiting HCPs (outreach visits) at their units to educate providers about PCC and operationalisations.  **Exclusion criteria:** Exclude statements about education where HCPs and stakeholders leave their unit to participate in educational meetings and code to 74. Provide stakeholders with time to attend educational meetings. | MI course  Log unit 4. |
| **19. Conduct ongoing training**  Plan for and conduct training in the clinical innovation in an ongoing way. | **Inclusion criteria:** Include statements indicating training that is ongoing, i.e., more than once.  **Exclusion criteria:** Exclude statements indicating training that only happens once or is related to educational outreach visits and code to 16. Conduct educational outreach visits. | Daily dialogue, reflection, and support for the implementation of team rounds.  Log unit 5. |
| **20. Create a learning collaborative**  Facilitate the formation of groups of providers or provider organizations and foster a collaborative learning environment to improve implementation of the clinical innovation. | **Inclusion criteria:** Include statements indicating that stakeholders and/or HCPs are collaborating within the region or nationally.  **Exclusion criteria:** Exclude statements where HCPs have been given protected time to develop or support one another in teams and code instead to 48. Organize clinician implementation team meetings. | Quality circle, increase knowledge about what PCC is and gain consensus among professions, doctors, nurses, nursing aids and management. Log unit 1.  We also try to get them to land in the same context to help each other. That was the idea with units XX and YY, who came together at the same workshop in the afternoon to connect and support each other. How do you do it? This is how we do it, yes, can you come and help us, and so on so that it can spread like ripples in a pond.  Dyadic interview unit DD. |
| **29. Develop educational materials**  Develop and format manuals, toolkits, and other supporting materials in ways that make it easier for stakeholders to learn about the innovation and for clinicians to learn how to deliver the clinical innovation. | **Inclusion criteria:** Include statements indicating that work has been focused on developing educational materials for stakeholders, HCPs, patients, or their next of kin.  **Exclusion criteria:** Exclude statements defining materials development for other purposes, i.e., reports to stakeholders, implementation plans and code to e.g., 77. Communication or 23. Develop a formal implementation blueprint. | [creation of a manual for all HCPs involved to work with the new daily round] Poster with our vision and accompanying abstract. A new suggestion for a daily round manual is created. Document from the specialist nurse’s diary at unit 6 |
| **31. Distribute educational materials**  Distribute educational materials (including guidelines, manuals and toolkits) in person, by mail, and/or electronically. | **Inclusion criteria:** Include all statements indicating that educational materials have been distributed.  **Exclusion criteria:** Exclude statements indicating that reminders to clinicians or sharing of information (e.g., progression of implementation) has been distributed and code to 58. Remind clinicians or 77. Communication. | Send information via mail on developed posters, abstracts and a video about the underpinnings for Open Dialogue to all managers who will forward the mail to all HCPs.  Log unit 6. |
| **43. Make training dynamic**  Vary the information delivery methods to cater to different learning styles work contexts, and shape the training in the innovation to be interactive. | **Inclusion criteria:** Include statements indicating that various methods have shaped information sharing, training, and education.  **Exclusion criteria:** Exclude statements indicating that information has been conducted without variation (e.g., formal lectures) and code to 15. Conduct educational meetings. | One of our strategies has been to appeal to as many people as possible. A lot of people want to see evidence and are triggered by that, and then some want more practical examples, so we try to see that as many as possible get what they need. This has been one of our starting points.  Dyadic interview unit DD. |
| ***74. Provide stakeholders with the possibility to attend educational meetings** | Provide stakeholders and HCPs with the possibility to attend educational meetings targeted toward different stakeholder groups (e.g., providers, administrators, other organisational stakeholders, and community, patient/consumer, and family stakeholders) to learn about the innovation.  **Inclusion criteria:** Include statements where stakeholders and HCPs have participated in educational meetings planned and conducted by others outside their unit.  **Exclusion criteria:** Exclude statements where the educational meetings have been orchestrated by their unit and code instead to 15. Conduct educational meetings. | Learning seminar  Log unit 5. |
| ***77. Communication** | Communicate with clinicians and stakeholders to exchange information about PCC and its implementation.  **Inclusion criteria:** Include statements indicating that one- or two-way communication has been conducted in face-to-face conversations, telephone- or net-based conversations, or relayed through other media such as e-mails or letters.  **Exclusion criteria:** Exclude statements indicating that communication was not the strategy's target and code instead to e.g., 31. Distribute educational materials or 58. Remind clinicians. | We really have these discussions, I think, all the time.  Dyadic interview unit 2.  We have it [pcc] included in our workplace meetings, and management group. So, it should be in all meeting protocols as a starting point. If we are going to keep this going, we need to somehow keep it going all the time in some way. So, we use both big and little strategies in some way.  Focus group unit 4. |
| 1. **Support clinicians** | | |
| **21. Create new clinical teams**  Change who serves on the clinical team, adding different disciplines and different skills to make it more likely that the clinical innovation is delivered (or is more successfully delivered). | **Inclusion criteria:** Include statements indicating that the team's composition has changed due to the addition of vocational roles.  **Exclusion criteria:** Exclude statements indicating that the team has changed due to revised roles and code instead to 59. Revise professional roles. Exclude statements indicating that new HCPs are employed with knowledge and skills in PCC and code instead to 76. Recruit clinicians with competence in innovation. | Stroke follow-up. Starting follow-up meetings for stroke patients who meet the entire team with four professionals. The idea is to have better follow-ups for the patients who get better information that is adapted by meeting different professionals (nurses, doctors, occupational therapists, and physiotherapists) and even more individualised because more professions can talk to the patient from different perspectives in comparison to how it was before with only one nurse.  Log unit 2. |
| **58. Remind clinicians**  Develop reminder systems designed to help clinicians to recall information and/or prompt them to use the clinical innovation. | **Inclusion criteria:** Include statements indicating that the core target of the strategy was to remind HCPs.  **Exclusion criteria:** Exclude statements indicating that reminding HCPs was not the core target of the strategy e.g., creating teams for reflections code instead to 48. Organize clinician implementation team meetings or 77. Communicate. | Personal health plan was a point of reminder at workplace meeting.  Log unit 5.  We have now decided that we will have this here, and we will continue with it. And if we are going to continue with it, then we must have it on the agenda [reminder about PCC at all meetings that take place at the health care unit]  Focus group unit 4.  We created magnetic buttons for the personnel, that today you get a button, for admissions [listening to and documenting the narrative when new patients are admitted]. If we discharge someone today and accept someone new, then it’s you who will do that in the afternoon. It is all about understanding. But then I think that the specific in this situation was the small magnetic buttons that said you have to.  Dyadic interview unit 1. |
| **59. Revise professional roles**  Shift and revise roles among professionals who provide care, and redesign job characteristics. | **Inclusion criteria:** Include statements indicating that roles have been revised or shifted among HCPs.  **Exclusion criteria:** Exclude statements indicating that new HCPs are added to the team and code instead to 21. Create new clinical teams. | Oh, she makes sure [Introduction of a coordinating nurse] there are plans for the relatives and the patients. Something no one really had responsibility for earlier.  Dyadic interview unit 2.  [Have created more time for the nurses by bringing in someone to run the kitchen]  Yes, what XX said there, if I, as a carer, have been responsible for a group session or followed my patient to a meeting with the doctor, then I can continue this work, so to say, the next day. And devote my time to PCC. The cook may not personally conduct, by definition, PCC, but it creates more possibilities for me to be person-centred.  Focus group unit 5. |
| ***75. Provide stakeholders with resources.** | Support clinicians/change agents implementing the innovation and give them protected time to reflect and learn on an individual basis about the implementation effort  **Inclusion criteria:** Include statements indicating that protected time has been given to individuals to reflect and learn about PCC or its implementation.  **Exclusion criteria:** Exclude statements indicating that team members have been given protected time to reflect and learn about PCC or its implementation code instead to 48. Organize clinician implementation team meetings. | Compilation of educational planning days. Presentation to the management group and all personnel.  Log unit 4. |
| ***76. Recruit clinicians with competence in the innovation** | Create job advertisements to profile the innovation and ask about clinicians’ competence when they apply for a position. Recruit HCPs who match the profile.    **Inclusion criteria:** Include statements indicating that recruitment was actively targeting HCPs with competence in PCC.  **Exclusion criteria:** Exclude statements indicating that the recruiting is especially targeting leaders for the change effort and code to 57. Recruit, designate, and train for leadership. Exclude statements indicating that new disciplines are added to a team composition and code instead to 21. Create new clinical teams. | Then we also have this in the advertisements, that we have a person-centred approach, that it’s a natural part of our work or whatever I have written, …And so there is a possibility here to, already in the interview, raise this, are you interested in this [PCC]? This is how we work here; this is a prerequisite for you to…  What do you know about this, and what do you think about this?  Dyadic interview unit 2. |
| 1. **Engage consumers** | | |
| **41. Involve patients/consumers and family members**  Engage or include patients/consumers and families in the implementation effort. | **Inclusion criteria:** Include statements indicating that patients have been included in the implementation effort.  **Exclusion criteria:** Exclude statements indicating that patients have been included to give feedback on the implementation effort (e.g., a patient survey about the perceived level of PCC) and code to 46. Obtain and use patients/consumers and family feedback. | Planning for the next learning seminar with a patient and another change agent.  Log unit DD.  For the patient’s sake, we are there, and we realise that it is their story that sticks, that makes an impression and that you take home with you. [change agent talking about patients being part of the learning seminars]  Dyadic interview unit DD. |
| **50. Prepare patients to be active participants**  Prepare patients/consumers to be active in their care, to ask questions, and specifically to inquire about care guidelines, the evidence behind clinical decisions, or about available evidence-supported treatments. | **Inclusion criteria:** Include statements indicating that patients are targeted to be active participants in their care.  **Exclusion criteria:** Exclude statements indicating that patients participate in the implementation effort and code to 41. Involve patients/consumers and family members. Exclude statements indicating that the strategy is to target patient feedback and code instead to 46. Obtain and use patients/consumers and family feedback. | Preparation of anti-tobacco campaign [for a waiting room campaign that aims to raise awareness regarding health and lifestyle choices to check reduced kidney function]  Log unit 3. |
| **69. Use mass media**  Use media to reach large numbers of people to spread the word about the clinical innovation. | **Inclusion criteria:** Include statements indicating that the main target of the strategy is to spread information through the media.  **Exclusion criteria:** Exclude statements indicating that information is spread to specific stakeholders and HCPs and code instead to 31. Distribute educational materials or 77. Communicate about the innovation. | Interview media. Spread the region’s work with PCC. [Change agents were interviewed by media to describe the work to implement more PCC across the region],  Log unit DD. |
| 1. **Utilize financial strategies** | | |
| **1. Access new funding**  Access new or existing money to facilitate the implementation. | **Inclusion criteria:** Include statements indicating that money is directly accessed to facilitate the implementation or operationalisation/delivery of the innovation.  **Exclusion criteria:** Exclude statements related to funding not directly related to implementation or operationalisation/delivery of the innovation. | Decision about economy/budget  Log unit DD.  Our seminars are free, so it’s work time then, for those who are there like. Otherwise, everything else is free.  Dyadic interview unit DD. |
| 1. **Change infrastructure** | | |
| **11. Change physical structure and equipment**  Evaluate current configurations and adapt, as needed, the physical structure and/or equipment (e.g., changing the layout of a room, adding equipment) to best accommodate the targeted innovation. | **Inclusion criteria:** Include statements indicating that physical structure or equipment has been changed or added to support the innovation or it’s operationalisation.  **Exclusion criteria:** Exclude statements indicating that changes have been made to physical structure or equipment for other reasons than accommodating the innovation. | [Renovation of premises-training premises for the patients] Patients should be more responsible and be more independent in their training. It makes training possible at all hours of the day. The patients have previously been under-stimulated, especially in the evenings and on weekends.  Log unit 2.  We are building a “documentation room” in the middle of the inpatients' ward in order to work closely with the patients.  Log unit 2.  My first home dialysis patient who I sent home took one of these [Skype telephone] home. And straight away, when I asked her if she would consider and wanted one so she could ring us and film or show the machine if it was standing beeping and she didn’t know what to do and thought, God what do I do now? She said she thought it was very good and felt much safer going with this. And it has worked well.  Focus group unit 3. |
| **12. Change record systems**  Change records systems to allow better assessment of implementation or clinical outcomes. | **Inclusion criteria:** Include statements indicating that the health record system has been changed to reflect the innovation, including added search words and health plans for continued care.  **Exclusion criteria:** Exclude statements indicating that changes in the health record system have been made without targeting the innovation or assessing its implementation or other clinical outcomes related to the innovation. | We are also … a test group for a [health care] journal template that should support this [PCC]. One of the words in the journal template is the patient narrative, which you can use instead of anamnesis or together with anamnesis.  Focus group unit 4. |
| **13. Change service sites**  Change the location of clinical service sites to increase access. | **Inclusion criteria:** Include statements indicating that changes have been made to increase access to patients and their next of kin.  **Exclusion criteria:** Exclude statements indicating that changes of the location of clinical service sites have been made but not with the intent to increase access for patients and their next of kin. | Procurement of video conference [equipment] to increase the patients’ opportunities for swift care planning, making possible an earlier return home.  Log unit 1.  Introduction of home rehab so that patients can have the option of going home earlier with rehab at home.  Log unit 2. |
| ****44. Mandate change**  Have leadership declare the priority of the innovation and their determination to have it implemented. | **Inclusion criteria:** Include statements indicating that leadership has mandated the priority of the innovation and their determination to have it implemented.  **Exclusion criteria:** Exclude statements indicating that mandate change has not been stipulated. | She is also involved [Senior manager]. She says we should do this. And that is how I think it gives the most support. It’s clear what she wants us to do. It’s not like well or so. Instead, it’s a way of supporting it as well.  Dyadic interview unit 1. |
| *****78. Act as a role model** | Have leadership and stakeholders function as role models to other stakeholders and HCPs. Appraise others in line with the ethical underpinnings of the innovation.  **Inclusion criteria:** Include statements indicating that stakeholders stated how they aspired to appraise staff with a person-centred approach to change other stakeholders and HCPs’ behaviour.  **Exclusion criteria:** Exclude statements where it is not stipulated that stakeholders regarded themselves as role models. | I think like this also as a manager. I try to bring this [PCC] with me in my work. I believe that if I have a person-centred approach towards staff, then I think that it will also affect them in their meeting with the patient. I mean, how I approach my staff, basically that I make sure that I am person-centred. I hope that it will somehow spill over to their attitudes and thoughts about it [PCC].  Dyadic interview unit 2. |

*Is given to new strategies that emerged during the data analysis. **is given to strategies found in interviews or documents but could not be linked to any activity logs and thus were not included in quantitative calculations from the activity logs. ***is given to new strategies that emerged during data analysis but could not be linked to activity logs.

1. Powell BJ, Waltz TJ, Chinman MJ, Damschroder LJ, Smith JL, Matthieu MM, Proctor EK, Kirchner JE: **A refined compilation of implementation strategies: results from the Expert Recommendations for Implementing Change (ERIC) project**. *Implement Sci* 2015, **10**:21.

2. Waltz TJ, Powell BJ, Matthieu MM, Damschroder LJ, Chinman MJ, Smith JL, Proctor EK, Kirchner JE: **Use of concept mapping to characterize relationships among implementation strategies and assess their feasibility and importance: results from the Expert Recommendations for Implementing Change (ERIC) study**. *Implement Sci* 2015, **10**:109.
