# Supplementary material for: Tracking, naming, specifying, and comparing implementation strategies for person-centred care in a real-world setting: a case study with seven embedded units
Source: BMC Health Serv Res. 2022 Nov 24;22:1409. doi: 10.1186/s12913-022-08846-x (PMC9685853; doi:10.1186/s12913-022-08846-x)
Supplement: Supplementary file 3 — Additional file 3. Example of discrete strategies named by ERIC and specified using Proctor et al.’s recommendations. [file 12913_2022_8846_MOESM3_ESM.docx]

**Additional file 3.**  Example of discrete strategies named by ERIC and specified using Proctor et al.’s recommendations.

| Strategy characteristics | Example from unit 2 | Example from unit 4 | Example from unit DD |
| --- | --- | --- | --- |
| ERIC name and cluster | 21. Create new clinical teams  Cluster: Support clinicians | 65. Use an implementation advisor  Cluster: Develop stakeholder interrelationships | 52. Promote network weaving  Cluster: Develop stakeholder interrelationships |
| Actor | Change agents at the unit | Change agent at the unit | Change agent at the DD |
| Action | Starting up a new team in which patients with follow up after stroke get to meet HCPs representing four vocational roles. | Meeting with change agent at the DD to get support in planning implementation of PCC and educational activities at the unit. | National network meeting with other stakeholders across the country. Sharing knowledge, information, and research with stakeholders in similar positions across the country. |
| Action target | Physiotherapists, occupational therapists, medical doctor, nurses, and managers at the OT and PT departments. | Change agent at the DD  Conceptual target: increased knowledge | Conceptual target: Sharing knowledge and monitoring the outside world. |
| Temporality | 2018-10-10 to 2018-11-27 | 2016-09-30 | 2018-01-11 |
| Dose | 3 meetings with 10 persons = 100 hours | 1 hour | 1 hour |
| Outcomes affected | To increase the quality of care for patients at follow up after stroke. Enabling partnership between patients and HCPs based on different patients and vocational perspectives according to patients’ beliefs and wishes. The team is operational on 27/11 | Not mentioned | Not mentioned |
| Justification | In line with PCC | Not mentioned | Not mentioned |
