# Supplementary material for: Tracking, naming, specifying, and comparing implementation strategies for person-centred care in a real-world setting: a case study with seven embedded units
Source: BMC Health Serv Res. 2022 Nov 24;22:1409. doi: 10.1186/s12913-022-08846-x (PMC9685853; doi:10.1186/s12913-022-08846-x)
Supplement: Supplementary file 4 — Additional file 4. Summaries of enacted activities at the embedded units. [file 12913_2022_8846_MOESM4_ESM.docx]

**Additional file 4.**

| The DD  205 activity logs | In charge of and participating in meetings with stakeholders at various regional and national levels, e.g., managers, politicians, and quality developers, to support buy-in, build coalitions, promote network weaving, and learn more about PCC and its implementation.  Involved patient representatives early in the planning stage and had patients being part of the lecturing at learning seminars.  Conducted and invited all units in the region to take part in learning seminars and other educational initiatives.  The DD used feedback from participants at each learning seminar to tailor strategies for future learning seminars.  Searched for and identified, developed, and distributed educational materials used at learning seminars and shared with all units or HCPs who wanted to learn more about PCC and continue in line with similar local educational meetings at their unit.  The DD engaged in projects throughout the region to enable more PCC (e.g., changing the health record system to include the search word narrative and a health plan, and discussing scale-up towards other stakeholders such as people at the municipality). |
| --- | --- |
| Unit 1  14 activity logs | HCPs representing a mix of vocational roles took part in a quality circle carried out by a national institute to learn more about PCC.  A nurse conducted part of her university training to become a specialist nurse at the ward and introduced and trained HCPs in the concept of using the narrative to HCPs working in one of the multibed rooms of the ward.  Local educational meetings were conducted at the unit.  Coordination nurses were supported in team meetings and discussions to increase buy-in towards PCC and its operationalisation to the local context.  Feedback from patients about their work was enabled by using a letterbox in the ward where patients were encouraged to write down their experiences and perceptions of care.  Videoconference equipment was procured to allow the next of kin and other stakeholders to participate in meetings with the patient and HCPs at the ward. |
| Unit 2  27 activity logs | Local educational meetings were conducted for all HCPs at the unit.  Results from a national patient survey were used as feedback from patients.  Remodelling of the ward was conducted so work could be accomplished more consistent with PCC (e.g., single rooms were thought to facilitate undisturbed meetings between HCPs and patients and conference rooms were built to have meetings catering for teams of HCPs to meet with patients and their next of kin to discuss such matters as rehabilitation plans).  New clinical teams with increased vocational roles were created to improve follow-up for stroke patients.  Professional roles were revised to increase continuity of care for patients.  Service sites were increased through home assessments for patients with memory loss.  Adaptability of PCC was promoted through home rehabilitation and increased treatment options for patients (e.g., horse rehabilitation). |
| Unit 3  70 activity logs | HCPs were enrolled in learning seminars every year across the implementation period.  Local educational meetings were conducted at the unit with similar take as the learning seminars, i.e., education about PCC, including role-plays, patient representatives, and staff from other units who shared their understanding and experience from working and implementing PCC.  A group of change agents represented by all care specialities was put together with the senior and junior managers to increase buy-in and spread.  Meetings were held to involve all HCPs in the workplace by having staff discuss how to adapt PCC to their local context and then vote for changes that should be prioritised.  All HCPs were enrolled in MI training to help HCPs identify patient resources  Increased access through videoconference equipment permitting contact with HCPs for home dialysis patients and contact with MD for patients in need of MD in the satellite unit. |
| Unit 4  38 activity logs | A group of change agents were assembled with the senior manager and junior managers, who represented the three subdivisions in the primary care unit.  A quality developer at the unit supported change agents to drive the change and engaged in projects throughout the region to allow for more PCC (e.g., working towards changing the health record system to include the search word narrative and a health plan).  Educational meetings were conducted at the unit to increase knowledge and support more robust team building.  All HCPs were enrolled in MI training, whereby a trainer came to the location to help HCPs identify patient resources. |
| Unit 5  29 activity logs | Educational meetings were conducted at the unit to increase knowledge and support buy-in.  After a new daily round had been successfully introduced for HCPs belonging to unit 6, the same work was carried out for HCPs working in teams belonging to unit 5.  Together with the frontline manager, a coordination nurse helped support the introduction of the new team round.  Roles were revised so that nursing aids took on more responsibility for contacting patients and their next of kin/social security. |
| Unit 6  30 activity logs | Educational meetings were conducted at the unit to increase knowledge and support buy-in.  Two nurses conducted part of their training to become specialist nurses at the ward and trained other HCPs belonging to unit 6 to change the daily round. They worked daily for a month to train HCPs and support the change at their unit.  Roles were revised so that nursing aids took on a larger responsibility for contacting patients and their next of kin/social security. |
